# TGF-β1 stimulates epithelial–mesenchymal transition and cancer-associated myoepithelial cell during the progression from in situ to invasive breast cancer

**DOI:** 10.1186/s12935-019-1068-7

**Published:** 2019-12-19

**Authors:** Li Wang, Cong Xu, Xia Liu, Yang Yang, Lu Cao, Guomin Xiang, Fang Liu, Shuling Wang, Jing Liu, Qingxiang Meng, Jiao Jiao, Yun Niu

**Affiliations:** 1 Tianjin Medical University Cancer Institute and Hospital, National Clinical Research Center of Cancer, Key Laboratory of Breast Cancer Prevention and Therapy, Tianjin Medical University, Ministry of Education, Key Laboratory of Cancer Prevention and Therapy, West Huanhu Road, Ti Yuan Bei, Hexi District, Tianjin, 300060 China; 2 The Second Department of Breast Cancer, Tianjin Medical University Cancer Institute and Hospital, Tianjin, 300060 China; 3 Department of Breast Cancer Pathology and Research Laboratory, Tianjin Medical University Cancer Institute and Hospital, Tianjin, 300060 China; 4 Department of Breast Oncology, Tianjin Medical University Cancer Institute and Hospital, Tianjin, 300060 China; 5 Department of Oncology, General Hospital of Tianjin Medical University, 154 Anshan Road, Heping District, Tianjin, 300052 China

**Keywords:** DCIS, Progression, Epithelial–mesenchymal transition, Myoepithelial cell, TGF-β1

## Abstract

**Background:**

The progression of ductal carcinoma in situ (DCIS) into invasive ductal carcinoma (IDC) is prevented by normal breast myoepithelial cells. Studies have suggested that EMT-associated genes were enriched in IDC in contrast to DCIS. This paper explored the relationship and potential mechanism between myoepithelial cells and EMT-associated genes in facilitating the transformation from DCIS to breast cancer.

**Methods:**

EMT markers and myoepithelial phenotypic markers in IDC, DCIS, and healthy breast tissue were characterized using immunohistochemical assay. Both in vivo and in vitro models were created to mimic the various cell–cell interactions in the development of invasive breast cancer.

**Results:**

We found that EMT markers were more abundant in invasive carcinomas than DCIS and adjacent normal breast tissue. Meanwhile, TGF-β1 regulated the morphology of MCF-7 (epithelial cells substitute) migration and EMT markers during the transformation from DCIS to invasive breast cancer. Additionally, TGF-β1 also regulated invasion, migration and cytokines secretion of MDA-MB-231 (myoepithelial cells substitute) and epithelial cells when co-cultured with MCF-7 both in vitro and in vivo.

**Conclusions:**

In conclusion, these findings demonstrated that both EMT phenotypes and cancer-associated myoepithelial cells may have an impact on the development of invasive breast cancer.

## Introduction

Ductal carcinoma in situ (DCIS) is recognized as a localized tumor cell proliferation in the ductal-lobular system that does not penetrate the basement membrane and has the potential to transform into invasive breast cancer [1]. The cascade of events that occur between benign and malignant transformation has not been sufficiently clarified and is a complex process dependent of both the microenvironment as well as the tumor cell properties [2, 3].

One such process that is known to be involved in carcinogenesis is the epithelial–mesenchymal transition (EMT). EMT occurs when epithelial cells acquire mesenchymal properties such as cytoskeleton reorganization, loss of cell polarity and breakdown of cell junctions—all of which lead to increased cell motility [4, 5]. Besides carcinogenesis, this process has also been demonstrated in tissue regeneration and wound healing [6]. Both local and disseminated tumor metastasis have been thought to be a product of the EMT, as this process bestows otherwise benign cells with the properties to escape the rigid constraints of the surrounding tissue architecture, such as the basement membrane. This process was instigated as a result of several extracellular stimuli of which transforming growth factor-β (TGF-β) played a predominant role [7–9]. Recent literature has documented an increase in EMT-related gene expression in invasive cancer in comparison to DCIS [10, 11]. Nevertheless, data on the expression of EMT markers in DCIS and invasive carcinoma is scarce.

Normal mammary gland physiology and development are highly dependent on myoepithelial cells which surround mammary ducts and lobular acini [12, 13]. These cells possess properties that naturally act to suppress tumor formation such as the ability to maintain epithelial cell polarity, providing a physical barrier between epithelial cells and the surrounding stroma and ensuring the integrity of the ductal-lobular basement membrane [14]. Nevertheless, the functional and phenotypical differences between normal breast tissue myoepithelial cells and DCIS-associated myoepithelial cells in the context of malignant transformation are not known. A majority of literature on the topic have instead focused more on luminal epithelial cells, although a number of molecular studies have suggested that there are differences between normal breast tissue myoepithelial cells and DCIS-associated myoepithelial cells that may be underlie latter’s propensity for malignant transformation [15, 16].

The current investigation explores the expression of EMT markers (N-cadherin, Snail, Twist, Vimentin, Zeb1, E-cadherin) in invasive carcinomas and DCIS. The functional and immunophenotypic characteristics of DCIS-associated myoepithelial cells were also assessed through myoepithelial cell phenotypic markers (Calponin, SMA, p63). Subsequent investigation showed that stimulation with TGF-β1 induced EMT in MCF-7. Cell-based assays were carried out to document the cascade of cell–cell interaction during the evolution from non-malignant to malignant. We originally used this co-culture system and other methods to demonstrate the TGF-β1 role between epithelial and myoepithelial cells in development of pre-invasive breast cancer both in vitro and in vivo. All the resulting experimental data indicated that TGF-β1 has a significant role in the transformation from premalignant to invasive breast cancer.

## Materials and methods

### Patient samples and clinical profiles

116 and 88 cases of formalin-fixed and paraffin-embedded surgical samples of breast IDC and DCIS respectively chose between 1 January 2004 and 31 December 2006 from patients treated in the Tianjin Medical University Cancer Institute and Hospital. This series is significant as it comprises a large cohort of patients under long-term monitoring in a single institution. All patients were women between the ages of 25 and 82 years (average of 48 years). Table 1 depicts other clinical characteristics. None of these patients had undergone neoadjuvant chemotherapy. Three pathologists (Yun Niu., Xiaolong Feng, and Shuhua Lv.) were involved in reviewing the histopathological results and diagnoses in accordance to the World Health Organization criteria. All patients provided written informed consent prior to any surgical procedures and sample collection. The study protocol was reviewed by the Human Ethical Committee of Tianjin Medical University Cancer Institute and Hospital.

**Table 1:** Clinical information for invasive ductal carcinoma (IDC) and ductal carcinoma in situ (DCIS)

Characteristic	DCIS (n = 88)		IDC (n = 116)	
	No.	%	No.	%
Age at diagnosis (years)				
≤ 50	46	52.3	80	69
> 50	42	47.7	36	31
Tumor size (cm)				
≤2	27	30.7	35	30.2
2–5	58	65.9	69	62.3
>5	3	3.4	12	7.4
Histological grade				
1			20	17.2
2			77	66.4
3			19	16.4
Nuclear grade				
1	24	27.3		
2	50	56.8		
3	14	15.9		
Lymph node status				
0	88	100	55	47.4
1–3	0	0	37	31.9
4–9	0	0	12	10.3
≥10	0	0	12	10.3
ER status				
Negative	20	22.7	50	43.1
Positive	68	77.3	66	56.9
PR status				
Negative	23	26.1	62	53.4
Positive	65	73.9	54	46.6
Her-2				
Negative	56	63.6	70	60.3
Positive	32	36.4	46	39.7
ki67				
<20	65	73.9	20	17.2
≥20	23	26.1	96	82.8

### Immunohistochemistry

4 μm thick slices of paraffin-embedded tissues were first sectioned. Paraffin was then removed from these sections before they were rehydrated and exposed overnight to primary antibodies at 4 °C. A broad-spectrum secondary antibody was then added onto these slices before treatment with DAB. Visualization of the final product with a light microscope was done after hematoxylin counterstaining. Immunostaining-based scores were done by three pathologists who were blinded to the clinical background of each patients’ sample. The location, intensity and percentage of immunoreactivity, were evaluated for each antibody. Staining intensities were graded from a scale of zero to three: (0, no reactivity; 1, weak reactivity; 2, moderate reactivity; 3, strong reactivity) while positive cell percentage were grouped into four cohorts: 0 (no tumor cells), 1 (1–25%), 2 (26–50%), 3 (51–75%), 4 (> 76%). The sum of grades of staining intensity and percentage of positive cells were (0–2, negative vs. 3–7, positive) for E-cadherin, N-cadherin, Snail, Twist, Vimentin, Zeb1, SMA, p63, and Calponin. Ki67 status was illustrated in terms of percentage of positive cells, with a threshold of 20% of positive cells, ER and PR were interpreted as positive if more than 1% of tumor cells demonstrated positive nuclear staining and whole membrane strong staining of HER2 + was determined when more than 10% of the tumor cells were strongly stained.

### Cell co-culture conditions and treatments

**Fig. 1:**
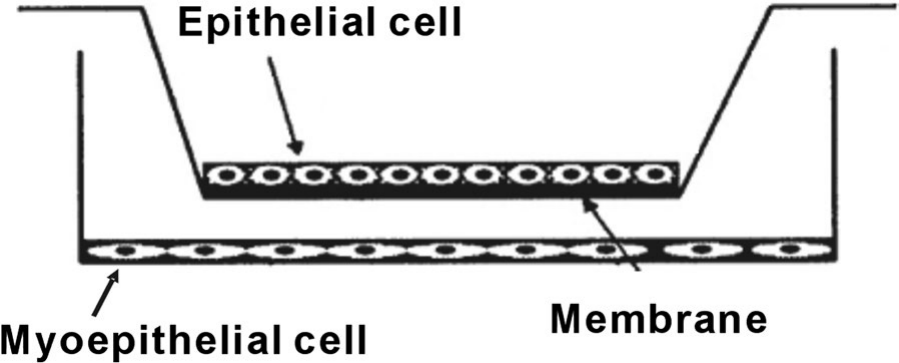
Experimental model. Epithelial cell and myoepithelial cell were co-cultured in the Transwell insert system

The Cell Bank of the Chinese Academy of Sciences (Shanghai, China) provided both cell lines MDA-MB-231 and MCF-7 which were cultured in RPMI-1640 mixed with 100 µg/ml each of streptomycin and penicillin as well as 10% fetal bovine serum. For investigating the paracrine effects of the cells, we used the Transwell insert system (Fig. 1). This system comprises of two compartments that were divided by a semi-permeable fenestrated membrane with 0.4 μm pore sizes (BD Biosciences, USA). The lower compartment contained MDA-MB-231 cells (3 × 10^5^ cell/per well, in 6-well plates) while the upper chamber contained MCF-7 cells (3 × 10^5^ cell/per well, in 6-well plates). These cells were co-cultured for 3 days under a humidified condition with 5% CO_2_ and at a temperature of 37 °C.

Our experiments consisted of three separate cell cohorts: MDA-MB-231 cells alone (MDA-MB-231 + Ctrl), MCF-7 + MDA-MB-231 cells (Co-culture + Ctrl), and MCF-7 + MDA-MB-231 cells + TGF-βl 10 ng/ml (Co-culture + TGF-βl). MCF-7 cells were subjected to a 24-hour exposure to TGF-β1 at 10 ng/ml prior to co-culture with MDA-MB-231 cells. TGF-β1 (100-21) was purchased from Pepro Tech (USA).

### Western blot

Cells were rinsed thrice with PBS before undergoing RIPA buffer and 1 mM PMSF- facilitated cell lysis. The resultant substrate was centrifuged and Western blot analysis was carried out on the supernatant fractions. These cell proteins were mixed into a SDS-PAGE membrane with 10% gel and subjected to electrophoresis. Protein bands in the gel were then immunoblotted onto polyvinylidene difluoride (PVDF) membranes before being probed with the aforementioned primary antibodies followed by secondary antibodies. ECL regents were used to detect positive bands of the blot.

### Immunofluorescence assay

Immunofluorescence assay was carried out as previously described [17]. CD10 (Bioss, China) CK8/18 (Thermo Fisher) were utilized for study. Immunofluorescence assays were visualized using Goat anti-Rabbit Alexa Fluor^®^ 594-conjugated (Zhongshan gold bridge) or Goat anti-Mouse Fluorescein–conjugated (Zhongshan gold bridge) antibodies. Cell nuclei were counterstained using DAPI (Thermo Fisher Scientific). Representative images were captured and analyzed by microscopy.

### RNA extract and qRT-PCR

Trizol reagent (Takara, Japan) was used to isolate total RNA from cell cultures in compliance to manufacturer’s protocols. Optical density (A_260_) methods were used to quantify RNA prior to its storage at − 80 °C until further usage. The Reverse Transcription System (TAKARA, Dalian, China) using oligo (dT) primers allowed for reverse transcription of RNA into cDNA. β-actin was used as a housekeeping gene to verify cDNA integrity. The single-stranded cDNA was amplified using the following primers:E-cadherin: Forward:5′-TCCATTTCTTGGTCTACGCC-3′, Reverse:5′-CACCTTCAGCCATCCTGTTT-3′; N-cadherin: Forward:5′-ACAGTGGCCACCTACAAAGG-3′, Reverse: 5′-CCGAGATGGGGTTGATAATG-3′; Vimentin: Forward: 5′-CCTTGAACGCAAAGTGGAATC-3′; Reverse: 5′-GACATGCTGTTCCTGAATCTGAG-3′; β-actin Forward: 5′-TCGTGCGTGACATTAAGGAG-3′; Reverse: 5′-ATGCCAGGGTACATGGTGGT-3′. β-actin was used as an internal control. The relative gene expression was calculated by the 2^−ΔΔCt^.

### MTT assays

Cell proliferation assay was carried out with the 3-(4,5-dimethylthiazol-2-yl)-2,5-diphenyltetrazolium bromide (MTT) assay. Each well of a 96-well plate were seeded with 5 × 10^3^ cells. The cells first underwent primary incubation before being incubated for 4 h with 20 μl MTT at 37 °C. The medium was then rinsed out and 150 μl DMSO was used to dissolve precipitated Formosan. A micro-plate auto-reader (Bio-Rad, USA) was used to detect absorbance at 490 nm.

### Wound healing assay

3 × 10^5^ cells were seeded in a 35 mm culture dish. A single scratch was inflicted with a 200 µl pipette tip on the cell monolayer (formed after allowing cells to proliferate for 24 h). The cells were imaged under phase-contrast microscopy at time zero and immediately post incision.

### Preparation of the conditioned medium

Media that was pre-conditioned in the Transwell insert systems were aspirated after 72 h, filtered through 0.2 μm pores and stored at − 80 °C.

### Transwell invasion assays

Transwell invasion assay was carried out in modified Matrigel-coated 24-well chambers. The lower chamber contained 3 × 10^5^ MCF-7 cells while the upper chamber contained 3 × 10^5^ MDA-MB-231 cells in serum-free medium. Cells were allowed to migrate during a 48-hour incubation period. Cells in the lower chamber with 10% were then counted across five randomly selected fields.

### ELISA

ELISA kit (R&D Systems) experiments were carried out using the conditioned media and were done in compliance to manufacturer’s instructions. The conditioned supernatants were collected from the Transwell co-cultures after 72 h of co-culture in order to determine the levels of levels of MCF-7 and MDA-MB-231 secreted cytokines, which included MMP-9 and IL-6. A microplate reader was used to measure the absorbance at 450 nm of each well. Standard curves were used to calculate the concentrations of chemokine protein concentrations.

### Xenograft mouse model

30, six weeks old female BALB/c nude mice weighing between 16 and 18 g each were purchased and feet in specific pathogen free (SPF) conditions. Procedures of animal care were passed by the institutional animal use and care committee of Tianjin Medical University Cancer Institute and hospital. Three independent groups of cells were treated as above. MDA-MB-231 cells alone (MDA-MB-231 + Ctrl), MCF-7 + MDA-MB-231 cells (Co-culture + Ctrl), and MCF-7 + MDA-MB-231 cells + TGF-βl 10 ng/ml (Co-culture + TGF-βl). 2 × 10^6^ MDA-MB-231 cells were administered into the fat pads of nude mice. Tumor volumes were measured at an interval of every 5 days post-tumor cells injection. All animals were sacrificed after 40 days and all tumors were collected and weighted. All tumors and livers, lungs were fixed in formalin for further hematoxylin–eosin staining (HE). Tumor volume was calculated using the formula: (length × width^2^)/2.

### Statistical analysis

The SPSS software (ver. 16; Chicago, IL, USA) was used to perform all statistical analyses. Immunohistochemical data were analyzed using χ^2^ test. Data are presented as mean ± standard deviation of three duplicated tests. Differences between groups were compared using Student’s t-test and one-way ANOVAS. Statistical significance was considered at *p* < 0.05.

## Results

### Expression of EMT markers in DCIS, IDC, and adjacent normal breast tissue

**Fig. 2:**
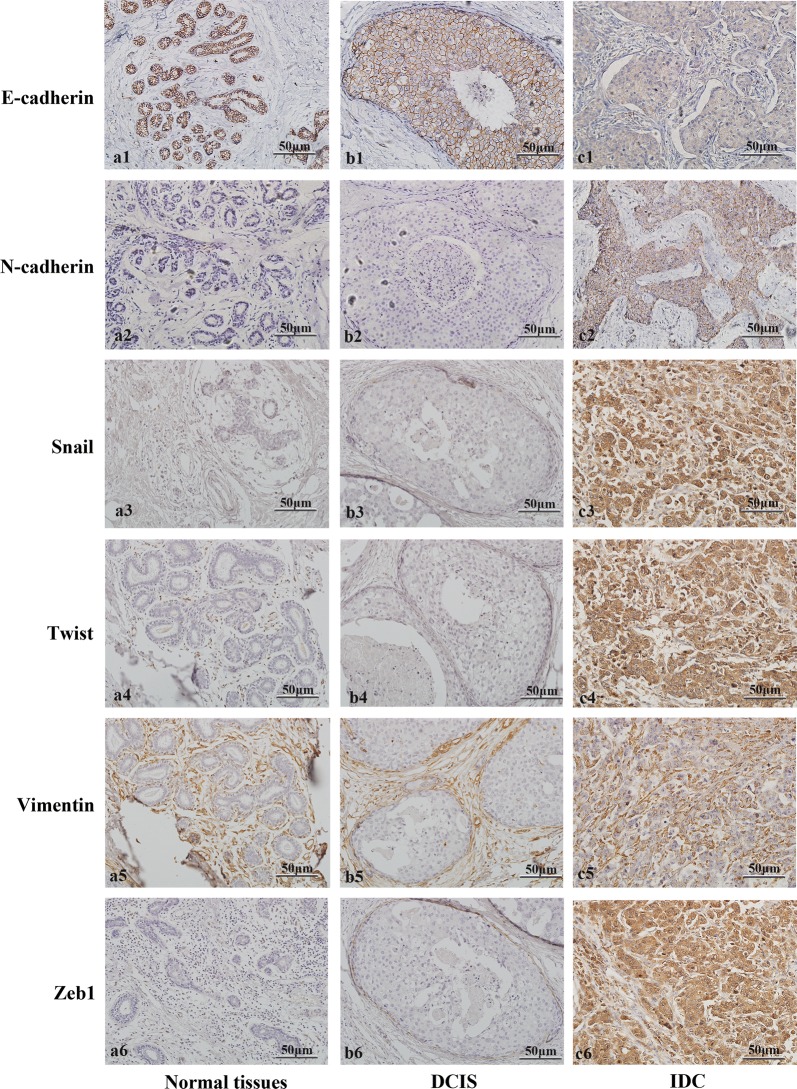
Expression of EMT markers in adjacent normal breast tissue, DCIS and IDC. **a1** Preserved membrane staining for E-cadherin in healthy breast tissue and DCIS (**b1**); **c1** loss of E-cadherin in IDC. **a2** Negative expression of N-cadherin in adjacent normal breast tissue and DCIS (**b2**); **c2** increased membrane N-cadherin expression is evident in IDC. **a3** Negative expression of snail in healthy breast tissue and DCIS (**b3**); **c3** increased cytoplasmic expression of Snail is evident in IDC. **a4** Negative expression of Twist in healthy breast tissue and DCIS (**b4**); **c4** increased cytoplasmic expression of Twist is evident in IDC. **a5** Negative expression of Vimentin in healthy breast tissue and DCIS (**b5**); **c5** increased cytoplasmic expression of Vimentin is evident in IDC. **a6** Negative expression of Zeb1 in healthy breast tissue and DCIS (**b6**); **c6** increased cytoplasmic expression of Zeb1 is evident in IDC. Original magnification× 200

**Fig. 3:**
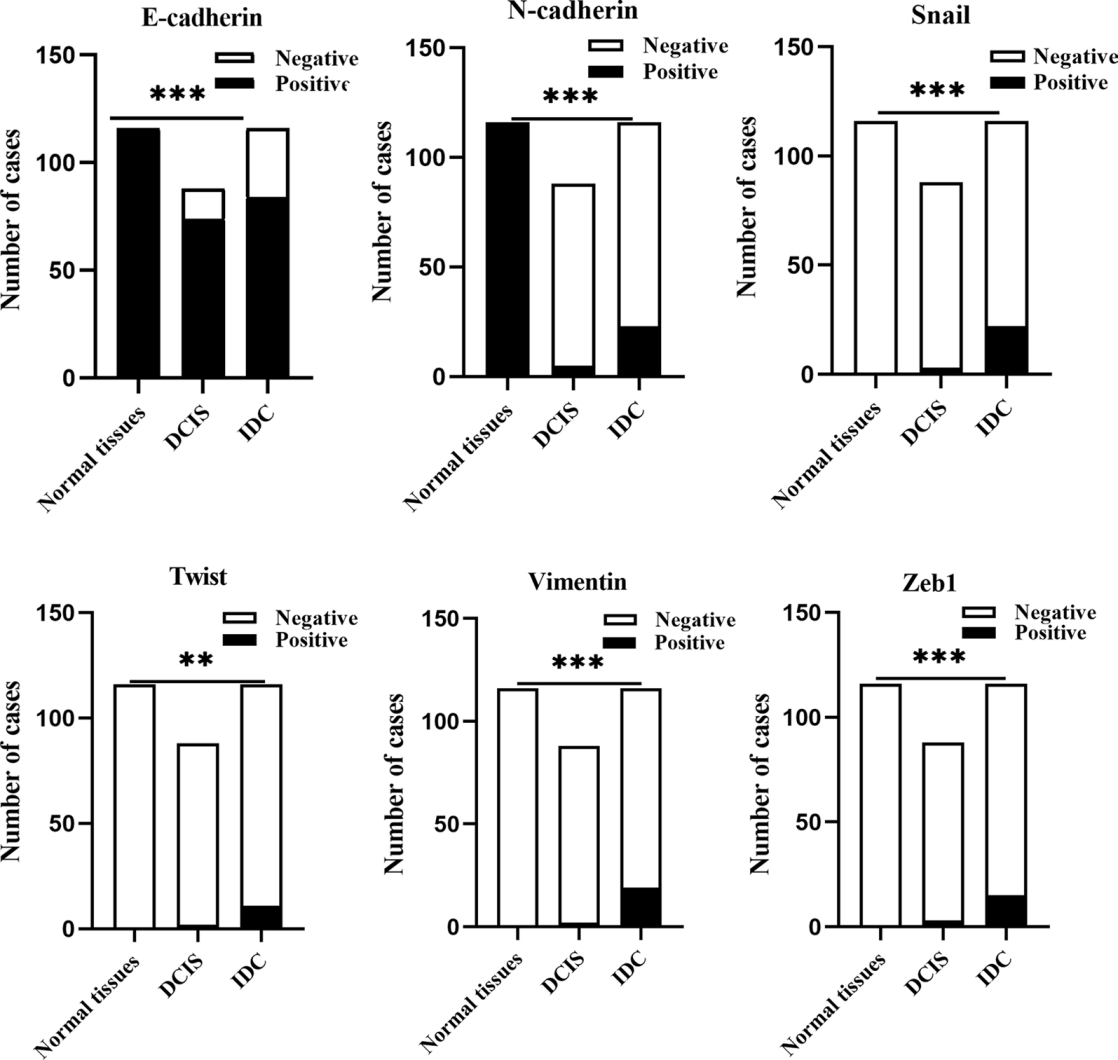
Expression of EMT markers in adjacent normal breast tissue, DCIS and IDC. **p < 0.01, ***p < 0.001

The loss of E-cadherin and increased expression of mesenchymal markers (N-cadherin, Snail, Twist, Vimentin, and Zeb1) were used as EMT indicators in breast cancer cells (Fig. 2). Invasive lobular carcinomas were not included in this study as they are characterized by loss of E-cadherin. N-cadherin, Snail, Twist, Vimentin, and Zeb1 were expressed in 19.8% (23/116), 19% (22/116), 9.5% (11/116), 16.4% (19/116),and 12.9% (15/116) of the invasive carcinomas. There was a loss of E-cadherin in 27.6% (32/116) of the invasive carcinoma samples (Fig. 3). In DCIS, N-cadherin, Snail, Twist, Vimentin, and Zeb1 were expressed in 5.7% (5/88), 3.4% (3/88), 2.3% (2/88), 2.3% (2/88), and 3.4% (3/88). E-cadherin loss was found in 15.9% (14/88) (Fig. 3). None of the adjacent normal breast tissue expressed N-cadherin, Snail, Twist, Vimentin, or Zeb1 and none of them displayed a loss of E-cadherin (Fig. 3).

The expressions of N-cadherin, Snail, Twist, Vimentin and Zeb1, and number of cells that demonstrated loss of E-cadherin were significantly higher in invasive carcinomas in contrast to normal tissue and DCIS samples (*p* < 0.05; Fig. 3).

### Phenotypic myoepithelial cell markers in adjacent normal breast tissue, DCIS and IDC

**Fig. 4:**
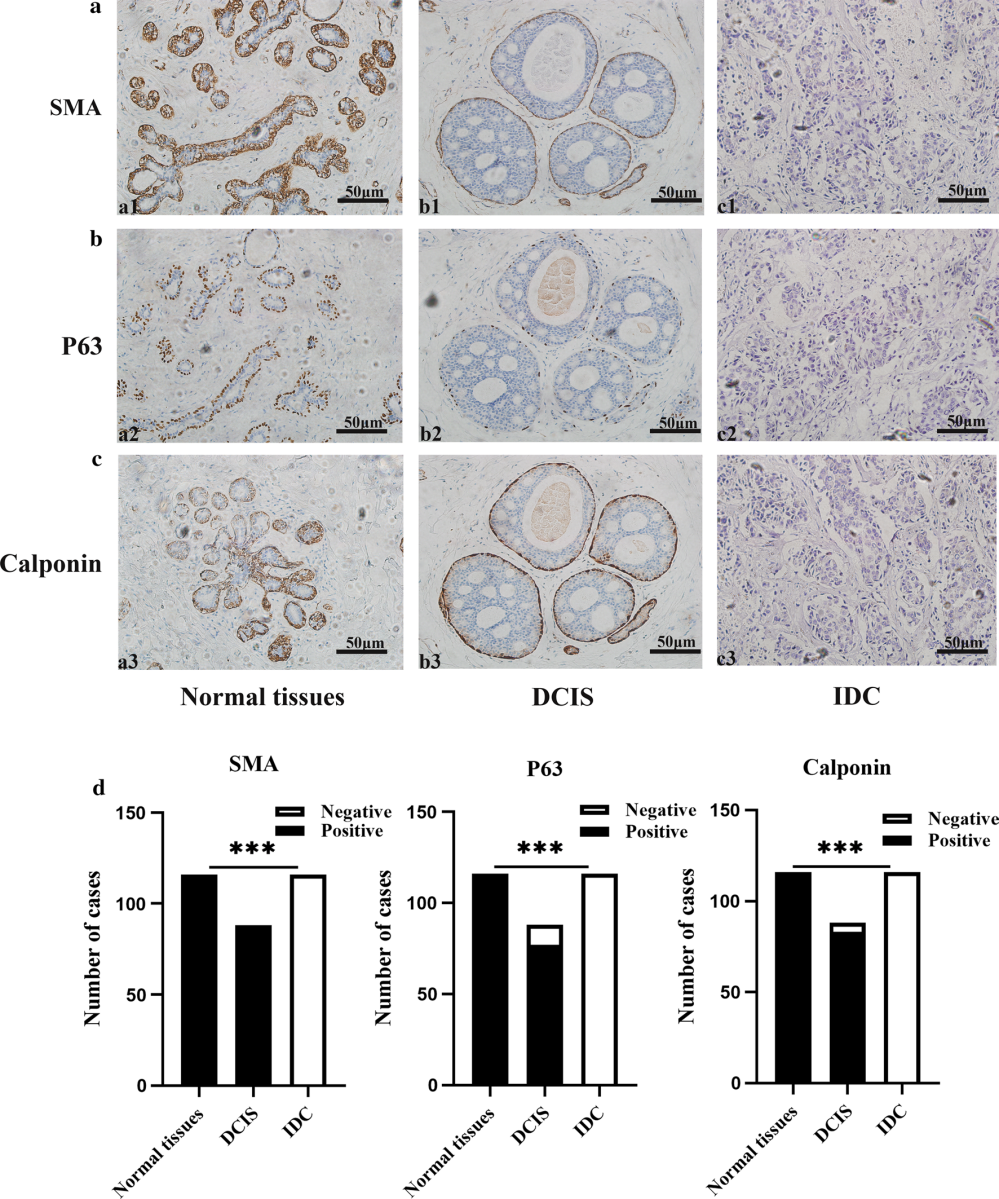
Myoepithelial cell marker expressions in adjacent normal breast tissue, DCIS and IDC. a1 SMA staining was continuously present in the myoepithelial cells layer and cytoplasm of the ductal-lobular system of adjacent normal breast tissue, and in the myoepithelial cell layer of DCIS (b1); Loss of SMA is evident in IDC (c1). a2 p63 stains were continuously expressed in the myoepithelial cell layer and nucleus of the ductal-lobular system of healthy breast tissue and was either discontinuously or absently expressed in the myoepithelial cell layer of DCIS (b2). Loss of p63 is evident in IDC (c2). a3 Calponin staining was continuously present in the myoepithelial cells layer and nucleus of the ductal-lobular system of healthy breast tissue, and in the myoepithelial cell layer of DCIS (b3); Loss of Calponin is evident in IDC (c3). Original magnification× 200. **d** Expression of myoepithelial cell markers in adjacent normal breast tissue, DCIS and IDC cases ***p < *0.001*

All one hundred and sixteen healthy breast tissue specimens (100%) and all eighty-eight DCIS (100%) specimens demonstrated strong SMA expressions in the myoepithelial cells (Fig. 4). However, none of the IDC samples expressed SMA (Fig. 4). All myoepithelial cells of 116 healthy normal breast tissue specimens demonstrated strong and continuous P63 expression (100%) (Fig. 4). In DCIS cells, p63 expression was continuous in 77/88 (87.5%) specimens, discontinuous in 5 (5.7%) specimens, and absent in 6 (6.8%) specimens. None of the IDC expressed p63 (Fig. 4d). Similarly, all healthy breast tissue samples demonstrated strong calponin expression (100%) (Fig. 4). 83 (94.3%) of DCIS specimens had significant Calponin expression, two (2.3%) samples had discontinuous calponin expressions and 3 (3.4%) specimens did not express calponin. None of the IDC expressed Calponin (Fig. 4d).

The differences in SMA, p63, and Calponin expressions in myoepithelial cells among healthy breast tissue, DCIS and IDC were statistically significant (*p *< 0.05, respectively; Fig. 4d).

### Identification of cell phenotype

**Fig. 5:**
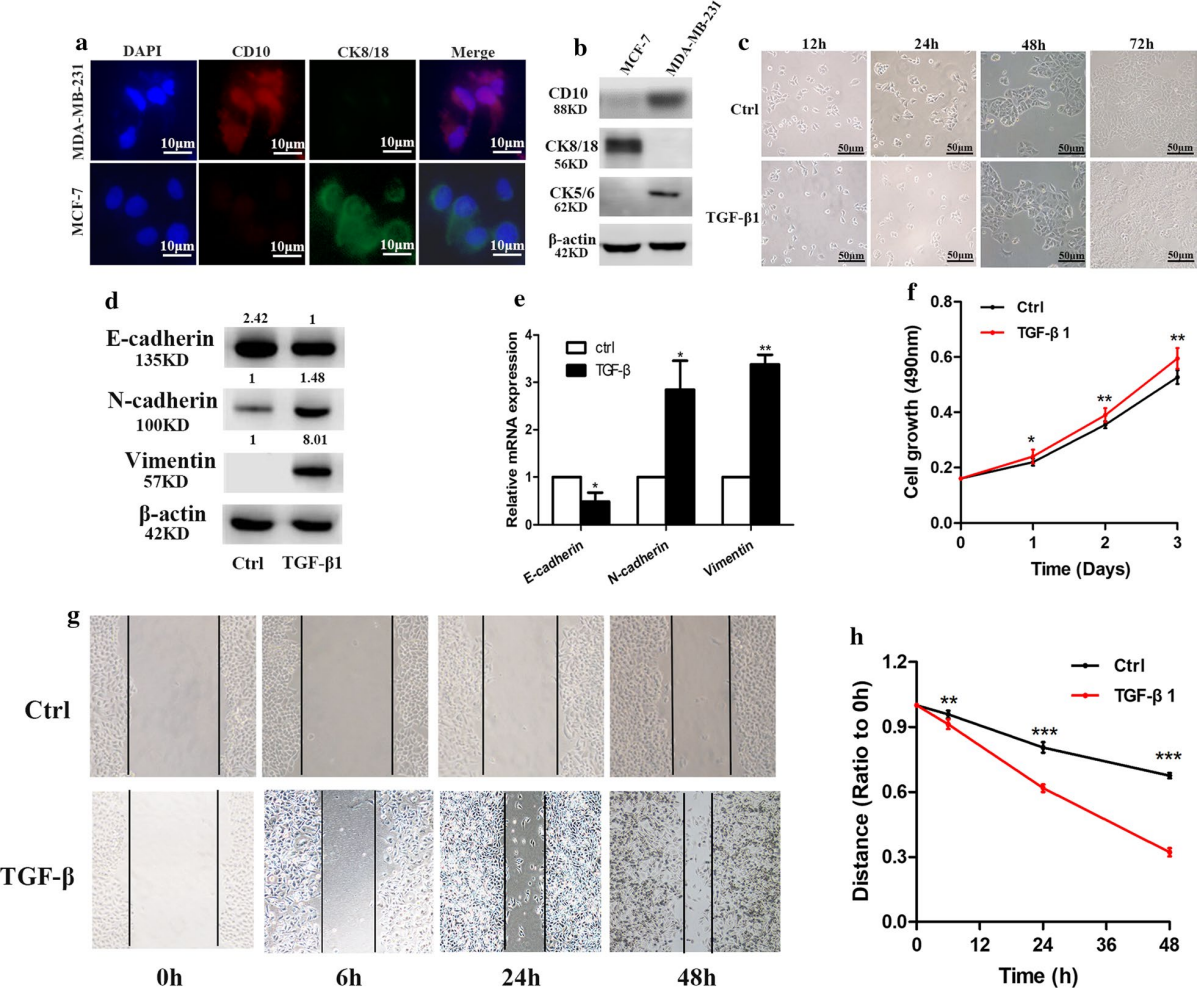
TGF-β1 regulates the morphology, migration and EMT markers of MCF-7 cells. **a** Immunofluorescence assay to detect CD10 and CK8/18 expression in MDA-MB-231 and MCF-7 cells. **b** Western blotting were used to quantify the CD10,CK8/18 and CK5/6 in MDA-MB-231 and MCF-7 cells. **c** TGF-β1 regulates MCF-7 cellular morphology. The bars are as indicated. **d** Western blots show the effect of TGF-β1 on Vimentin, N-cadherin and E-cadherin in MCF-7 cells. **e** qRT-PCR shows the effect of TGF-β1 on the mRNA levels of E-cadherin, N-cadherin and Vimentin in MCF-7 cells. **f** MTT assay assessed the effect of TGF-β1 on cell proliferation in MCF-7 cells. **g**, **h** Scratch wound-healing assay assessed the effect of TGF-β1 on the migration of MCF-7 cells. All data was showed as mean ± SD, *p < 0.05, **p < 0.01, ***p < 0.001

Breast cancer cell lines representing epithelial cells (MCF7) and myoepithelial cells (MDA-MB-231) were used in this study. Firstly, immunofluorescence assay and western blotting were used to quantify cell phenotype. MCF7 cells demonstrated no CD10 and CK5/6 but high levels of CK8/18, while the converse results were seen in MDA-MB-231 cells (Fig. 5a, b). These findings confirm the viability of MDA-MB-231 as a representative of myoepithelial cells and MCF7 as a representative of epithelial cells.

### TGF-β1 regulates the morphology, migration and EMT markers of MCF-7

MCF-7 cells demonstrated cell–cell adhesion and epithelial-like morphology. Upon exposure to TGF-β1, there was a change of cell morphology from oval to spindle shape as well as a decrease in cell–cell adhesion (Fig. 5c). Additionally, there was a reduction of E-cadherin at the protein and mRNA levels, and an increase in mesenchymal markers N-cadherin and Vimentin in MCF-7 expressions (Fig. 5d, e). The induced motility and migration of TGF-β1 were not a result of reduced cell viability as confirmed the cell proliferation (MTT assay) which demonstrated no change despite exposure to TGF-β1 (Fig. 5f). A wound-healing assay was used to determine the influence of TGF-β1 on cell migration. Stimulation with TGF-β1 in MCF-7 significantly enhanced the migration of cells in contrast to control cells (*p *< 0.05) (Fig. 5g, h). Taken together, TGF-β1 is a critical factor that regulates MCF-7 morphology, migration and expression of EMT markers during the progression from in situ to invasive breast cancer.

### TGF-β1 regulates migration, invasion and cytokines secretion of MDA-MB-231 when co-cultured with MCF-7

**Fig. 6:**
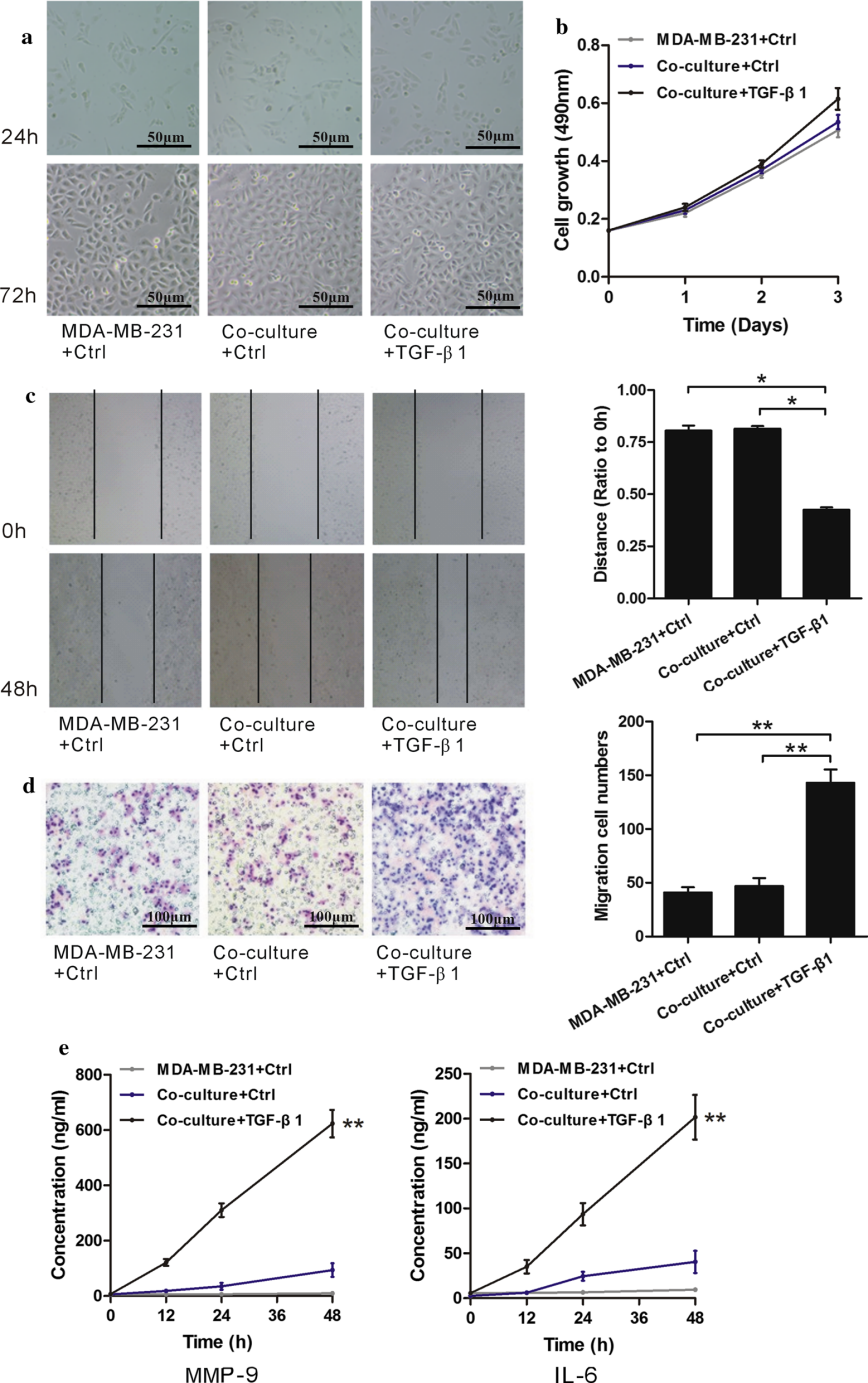
TGF-β1 regulates migration, invasion and cytokines secretion of MDA-MB-231 cells when co-cultured with MCF-7 cells. **a** MDA-MB-231 cellular morphology remained. The bars are as indicated. **b** MTT assay evaluated TGF-β1 effects on MDA-MB-231 cell proliferation when co-cultured with MCF-7 cells. **c** Scratch wound-healing assay assessed TGF-β1 effects of MDA-MB-231 cell migration when co-cultured with MCF-7 cells. **d** Transwell assay assessed TGF-β1 effects on the invasion of MDA-MB-231 cells when co-cultured with MCF-7 cells. **e** MMP-9 and IL-6 results obtained by the ELISA. All data was showed as mean ± SD, *p < 0.05, **p < 0.01, ***p < 0.001

MDA-MB-231 remained spindle shape when co-cultured with MCF-7 stimulated with TGF-β1 (Fig. 6a), while the MTT assay demonstrated no change despite stimulation with TGF-β1 (Fig. 6b). Co-culture + TGF-β1 group significantly enhanced the migration of cells compared with MDA-MB-231 alone and Co-culture + Ctrl group (*p* < 0.05) (Fig. 6c). In addition, we found similar results in Transwell assay (*p* < 0.05) (Fig. 6d). The Co-culture + TGF-β1 group produced higher levels of MMP-9 and IL-6 than the MDA-MB-231 alone and the Co-culture + Ctrl group (*p* < 0.05) (Fig. 6e). Co-culture + Ctrl and MDA-MB-231 + Ctrl produced an equal amount of MMP-9 and IL-6 when compared to levels produced by MCF-7 alone. Co-culture + Ctrl demonstrated the impact on MCF-7 on MDA-MB-231 in the co-culture system. We concluded that TGF-β1 regulates migration, invasion, and cytokines secretion of MDA-MB-231 throughout the evolution from in situ to invasive breast cancer when co-cultured with MCF-7.

### TGF-β1 effectively promoted tumor growth and metastasis when MDA-MB-231 co-cultured with MCF-7 compared with the other two groups

**Fig. 7:**
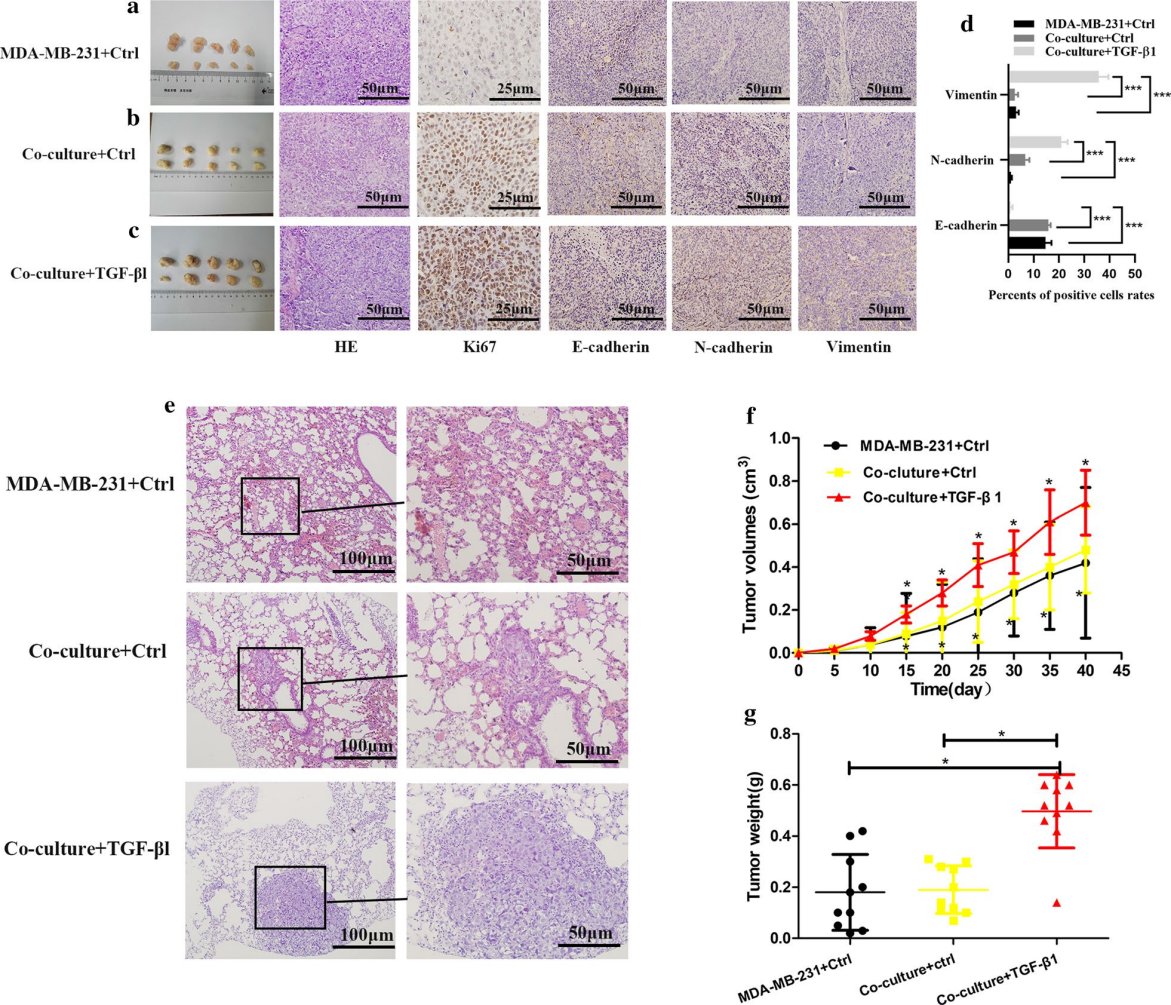
TGF-β1 effectively promoted tumor growth and metastasis when MDA-MB-231 co-cultured with MCF-7 compared with the other two groups. **a** The tumors, Hematoxylin–eosin staining and the representative staining images of Ki67, E-cadherin, N-cadherin and vimentin of mice tumors in MDA-MB-231 + Ctrl. The bars are as indicated. **b** The tumors, Hematoxylin–eosin staining and the representative staining images of Ki67, vimentin, N-cadherin and E-cadherin in mice tumors in Co-cluture + Ctrl. The bars are as indicated. **c** The tumors, Hematoxylin–eosin staining and the representative staining images of Ki67 vimentin, N-cadherin and E-cadherin of mice tumors in Co-culture + TGF-β1. The bars are as indicated. **d** The results of E-cadherin, N-cadherin and Vimentin expression in three groups. **e** Hematoxylin–eosin staining of mice lungs in MDA-MB-231 + Ctrl, Co-culture + Ctrl, Co-culture + TGF-β1. The bars are as indicated. **f** TGF-β1 increased the speeds of tumors growth in vivo when MDA-MB-231 cells co-cultured with MCF-7 cells. (*p < 0.05). **g** Statistical analysis of tumor weight in different groups. The values indicate the mean ± standard deviation, n = 10/group. All data was showed as mean ± SD, *p < 0.05, **p < 0.01, ***p < 0.001

To verify the function of TGF-β1 in vivo, three groups are conducted: MDA-MB-231 cells alone (MDA-MB-231 + Ctrl), MCF-7 + MDA-MB-231 cells (Co-culture + Ctrl), and MCF-7 + MDA-MB-231 cells + TGF-βl 10 ng/ml (Co-culture + TGF-βl) (Fig. 7a–c). Nude mice of Co-culture + TGF-βl were found to have much larger tumors than MDA-MB-231 + Ctrl and Co-culture + Ctrl (Left panels of Fig. 7a–c). There were significant differences in tumor volumes between MDA-MB-231 + Ctrl and Co-culture + Ctrl or Co-culture + TGF-βl (Fig. 7f) as validated by the weights of the individual tumors (Fig. 7g). However, there were no significant differences between Co-culture + Ctrl and MDA-MB-231 + Ctrl. Immunohistochemistry staining of Ki67, E-cadherin, N-cadherin, Vimentin of the tumors were also conducted. These results showed increased proliferation of Ki67 and enhanced Vimentin and N-cadherin expressions in Co-culture + TGF-βl compared with the other two groups (Fig. 7a–d). However, the converse was seen with regard to E-cadherin expressions. The HE slides of livers showed no metastasis in all groups, while lung metastasis was observed in Co-culture + TGF-βl and Co-culture + Ctrl. Besides, metastasis areas in co-culture + TGF-β1 was more larger than the co-culture + Ctrl group. No metastasis was seen in the remaining groups (Fig. 7e).

## Discussion

Mesenchymal cell exhibit augmented migratory and pro-carcinogenic phenotypes. This EMT process allows epithelial cells to lose their cell–cell adhesive qualities and acquire mesenchymal properties. It therefore comes as no surprise that the EMT process is thought to be a critical instigator of the transformation from in situ to invasive carcinoma [18]. Nevertheless, literatures supporting this theory are scarce, with only a few studies investigating the different EMT marker profiles between DCIS and invasive carcinomas. TGF-β1 and c-met expressions were raised during the evolution of DCIS to invasive carcinoma, as documented by Logullo et al. [19]. Knudsen et al. [10] has recently demonstrated an upregulation of myoepithelial cell-specific genes and EMT-associated genes in the epithelial cells of invasive carcinoma in comparison to DCIS, suggesting that molecular reprogramming of the epithelial cells are vital in triggering carcinogenesis. Expressions of SMA and N-cadherin, rates of β-catenin were all markedly raised as well as E-cadherin was reduced in invasive carcinomas when compared to pure DCIS [20]. In line with all these studies, our results also demonstrated that there was a significantly higher rate of E-cadherin loss and higher expressions of N-cadherin, Snail, Twist, Vimentin and Zeb1 in invasive carcinoma in comparison to DCIS.

While EMT has been implicated in tumor invasion partly by reducing cell–cell adhesion, the myoepithelial phenotype has been reported to increase cell motility [21]. Myoepithelial cells surrounding DCIS were found to possess significant epigenetic, genetic and molecular differences in comparison to those in the vicinity of normal breast lobules and ducts [22]. Significant genetic changes include an upregulation of genes for chemokines that augment epithelial cell invasion, migration and proliferation and a downregulation of genes controlling normal myoepithelial cell characteristics [14]. 13.3% of specimens of DCIS and all of the IDC samples in our study demonstrated no p63 staining, a finding that was also seen in Werling et al. who demonstrated a discontinuous p63 staining pattern in 10% of DCIS cases [23]. Myoepithelial cells associated with noninvasive proliferations, vascular smooth muscle and normal breast tissue myoepithelial cells all demonstrated high calponin expressions [24]. A study by Hilson et al. found that the myoepithelial cells of DCIS had suppressed calponin expressions [16], a finding that is mirrored in our study. Russell et al. reported that the earliest indication of a compromised myoepithelium was the loss of p63, followed by calponin loss at the intermediate stages [25], and SMA changes being the latest marker. These results lay the foundation for further research into the influence of myoepithelial cells on DCIS progression to invasive carcinoma.

TGF-β1 stimulation on MCF-7 allowed us to establish an EMT model, which in turn enabled us to demonstrate the EMT effect of MCF-7 on MDA-MB-231 in a co-culture system. TGF-β1 indeed regulates EMT though inhibition of MCF-7 cell motility and compromising cell–cell adhesion. The EMT is marked by an increase in mesenchymal markers (N-cadherin and Vimentin) and by a loss of epithelial markers (E-cadherin) [26]. These results mirror those of existing literature. TGF-β1 also regulated the migration of MCF-7, a feature that did not appear to depend on its general impact on cellular proliferation. To understand the in vitro mechanisms of epithelial cell and myoepithelial cell, a co-culture system allowing the interaction of myoepithelial and epithelial cells (a condition mimicking the tumor microenvironment) was created. This was done by using Transwell chambers, which allowed separation of cells via a porous membrane that allowed free diffusion of soluble factors. Martinez et al. recorded that medium conditioned with malignant epithelial cell was able to induce myoepithelial cell FGF2 secretion that supports carcinogenesis [27], Our results suggest that TGF-β1 increases MDA-MB-231 migration, invasion and cytokines secretion when co-cultured with MCF-7. Simultaneous silencing of MMP-9 in breast cancer cells decreased the adhesiveness, invasive, migratory and wound healing characteristics of cells [28]. Allinen et al. found that DCIS-associated myoepithelial cells up-regulate the synthesis of enzymes linked to the breakdown of ECM and BM such as MMPs [29]. In agreement with their study, our results suggested that MMP-9 secretion increased when MCF-7 stimulated with TGF-β1 and co-cultured with MDA-MB-231. Martinez et al. showed that myoepithelial cells produce IL-6 in in situ condition [30]. In particular, IL-6 levels peaked after a day period of cell culture. In agreement with their study, our results suggest that IL-6 secretion increases when MCF-7 stimulated with TGF-β1 and co-cultured with MDA-MB-231.

Although we have shown that stimulation with TGF-β1 bought about EMT in breast cancer, the exact domains and other molecules involved in this interaction remain to be clarified. Additional studies are necessary to further elucidate the effects of MDA-MB-231 on MCF-7 in the co-culture system. We conducted animal studies to further validate the in vivo role of EMT in the progression of in situ to invasive carcinoma. These results demonstrated that the existence of TGF-β1 in a MDA-MB-231 co-culture with MCF-7 resulted in EMT promotion, which in turn lead to increased tumor growth and metastatic capability to the lungs. Nevertheless, this co-culture system has its potential limitations, given the dynamics and complexity of epithelial cell–myoepithelial cell interactions in the in vivo microenvironment. While soluble factors are able to freely diffuse through the porous membrane of this system, direct cellular interaction is not present. Additionally, our system does not take into account the presence of other genetic mutations that may influence proliferation. These factors may also lead to the differences in cell survival across different co-culture groups.

## Conclusion

Taken together, our study demonstrated that there was an increase of mesenchymal marker expression as well as a higher rate of loss of myoepithelial phenotypic markers and loss of E-cadherin in invasive carcinomas when compared to normal healthy breast tissue and DCIS. Most importantly, TGF-β1 was critical in regulating the morphology, migration, and EMT markers of MCF-7.TGF-β1 regulates migration, invasion, and cytokines secretion of MDA-MB-231 when co-cultured with MCF-7 in vivo subcutaneous tumor formation and metastasis. These findings indicated that EMT phenotypes and cancer-associated myoepithelial cell may be present in the evolution of in situ to invasive breast cancer.

## Data Availability

Please contact the corresponding author for all data requests.

## Abbreviations

DCIS: ductal carcinoma in situ
IDC: invasive ductal carcinoma
TGF-β1: transforming growth factor-β
EMT: epithelial–mesenchymal transition
ECM: extracellular matrix
BM: basement membrane
FGF2: fibroblast growth factor 2
MMPs: matrix metalloproteinases
